# An ArsR Transcriptional Regulator Facilitates *Brucella* sp. Survival via Regulating Self and Outer Membrane Protein

**DOI:** 10.3390/ijms221910860

**Published:** 2021-10-08

**Authors:** Feijie Zhi, Dong Zhou, Jialu Chen, Jiaoyang Fang, Weifang Zheng, Junmei Li, Mingyue Hao, Yong Shi, Yaping Jin, Aihua Wang

**Affiliations:** 1 College of Veterinary Medicine, Northwest A&F University, Yangling 712100, China; zhifeijie@nwafu.edu.cn (F.Z.); zhoudong1949@nwafu.edu.cn (D.Z.); 2017050590@nwafu.edu.cn (J.C.); fangjiaoyang@nwafu.edu.cn (J.F.); 2019050547@nwafu.edu.cn (W.Z.); junmei_li@nwafu.edu.cn (J.L.); Gdk960101@nwafu.edu.cn (M.H.); 2019055318@nwafu.edu.cn (Y.S.); 2 Key Laboratory of Animal Biotechnology of the Ministry of Agriculture, Northwest A&F University, Yangling 712100, China

**Keywords:** *Brucella*, ArsR, Omp25D, metal ion homeostasis, virulence

## Abstract

The arsenic acid-resistant (ArsR) family transcriptional regulators are widely distributed in microorganisms, including in the facultative intracellular pathogen *Brucella* spp. ArsR proteins are implicated in numerous biological processes. However, the specific roles of ArsR family members in *Brucella* remain obscure. Here, we show that ArsR6 (BSS2_RS07325) is required for *Brucella* survival both under heat, oxidative, and osmotic stress and in a murine infection model in vivo. RNA-seq and ChIP-seq reveal that 34 potential target genes for ArsR6 protein were identified, among which eight genes were up-regulated and 26 genes were down-regulated, including outer membrane protein 25D (Omp25D). ArsR6 autoregulates its own expression to maintain bacterial intracellular Cu/Ni homeostasis to benefit bacterial survival in hostile environments. Moreover, ArsR6 also regulates the production of virulence factor Omp25D, which is important for the survival of *Brucella* under stress conditions. Significantly, Omp25D deletion strain attenuated in a murine infection model in vivo. Altogether, our findings reveal a unique mechanism in which the ArsR family member ArsR6 autoregulates its expression and also modulates Omp25D expression to maintain metal ion homeostasis and virulence in *Brucella*.

## 1. Introduction

*Brucella* is a facultative intracellular pathogen that is the causative agent of the important zoonotic infection brucellosis that induces a chronic, debilitating condition in humans and reproductive system disease in animals [1,2,3,4]. The arsenic acid-resistant (ArsR) family is distributed widely in prokaryotes, including *Brucella*, and regulates target gene expression at the transcriptional level [5,6]. The main function of ArsR family transcriptional regulators is as metalloregulatory proteins that induce metal ion uptake, efflux, sequestration, and detoxification to maintain intracellular metal ion homeostasis under extreme environmental conditions [5,6,7]. However, host cells may capitalize on both the toxicity and essentiality of metal ions to defend against intracellular pathogens [8]. In response, pathogens possess a variety of strategies to circumvent the ability of the host to restrict the use of metal ions, including free metal ion acquisition systems and metalloregulatory proteins [8,9]. *Brucella* has six ArsR family members, three of which are encoded by chromosome I and three of which are encoded by chromosome II [10]. However, the specific roles of ArsR family members in *Brucella* survival under heavy metal ions conditions and during intracellular infection remain unknown.

In addition to maintaining metal ion homeostasis, numerous studies have shown that ArsR family members are involved in diverse biological processes [6]. SdpR, CyeR, and SoxR are typical representative members of the ArsR family and mediate the response to hostile environments [6,11]. However, it is unknown whether ArsR family members in *Brucella* are involved in stress responses. In addition, certain ArsR family members influence bacterial virulence by regulating target gene expression. Mutation of certain transcriptional regulators, including ArsR family members, attenuates the virulence of *Brucella* in mice [10]. However, the mechanisms by which these ArsR mutants are impaired in intracellular survival remain unclear.

The aim of this work was to reveal *Brucella* BSS2_RS07325 (ArsR6) functions during infection. Here, we explored the role of ArsR family members ArsR6 in *Brucella* survival in vitro and in persistence of infection in vivo. We found that ArsR6 is a classical metalloregulatory protein that autoregulates its own expression and virulence factor Omp25D expression to enhance *Brucella* survival. These results lay the foundation for exploring the pathogenic mechanisms of Brucella.

## 2. Results

### 2.1. ArsR6 Is Required for Brucella Survival in a Mouse Model of Infection

**Figure 1:**
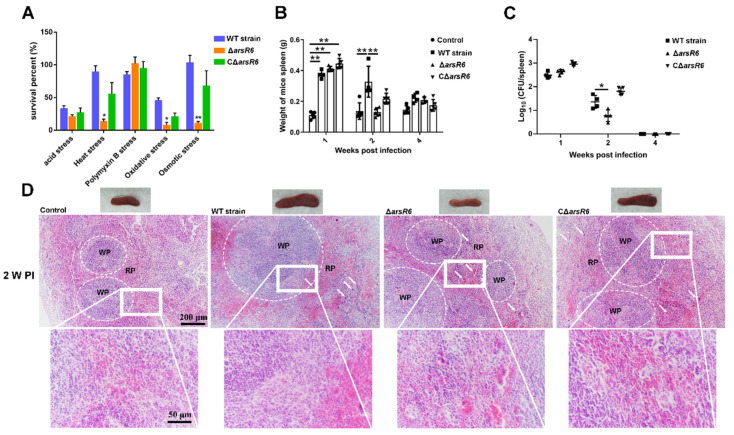
*Brucella* transcriptional regulator ArsR6 is required for bacterial survival during stress conditions and in mice. (**A**) The survival of bacteria under stress conditions. The WT, Δ*arsR6,* and CΔ*arsR6* strains were cultivated at pH 5.5 or 50 °C or were treated with polymyxin (10 μg/mL), H_2_O_2_ (0.5 mM), or sorbitol (0.5 M). Data represent mean and standard deviation of N = 3 (independent biological replicates). The asterisks indicate significant differences (** *p* < 0.01) based on two-way ANOVA followed by Tukey’s post hoc test of honestly significant differences (two-tailed). (**B**) Spleen weights were measured at 1, 2, and 4 weeks post-infection. The control represents uninfected BALB/c female mice injected with PBS. Data represent mean and standard deviation of N = 5 (independent biological replicates). The asterisks indicate significant differences (** *p* < 0.01) based on two-way ANOVA followed by Tukey’s post hoc test of honestly significant differences (two-tailed). (**C**) The bacterial load was measured in homogenates at 1, 2, and 4 weeks post-infection. Data represent mean and standard deviation of N = 5 (independent biological replicates). The asterisks indicate significant differences (* *p* < 0.05) based on two-way ANOVA followed by Tukey’s post hoc test of honestly significant differences (two-tailed). (**D**) Representative micrographs of the spleen histopathology at 2 weeks post-infection. Arrows, macrophages; WP, white pulp; RP, red pulp; square, magnification box.

An ArsR6 deletion strain and complemented strain were obtained successfully by PCR analysis (data not displayed) and RT-PCR analysis (Figure S1A,B). The growth curve of Δ*arsR6* was similar to that of the wild-type (WT) strain in TSB, indicating that the ArsR6 deletion had no effect on *Brucella* growth in these conditions (Figure S1C). The survival rate of Δ*arsR6* was decreased in heat, oxidative, and osmotic stress conditions compared to the WT strain, but was restored partly or fully in CΔ*arsR6* (Figure 1A). These data indicate that ArsR6 is required for the response to acid, oxidative, and osmotic stress conditions in *Brucella*.

Next, we assessed Δ*arsR6* intracellular survival in RAW264.7 macrophages. However, no significant difference was observed between the WT- and Δ*arsR6*-infected groups (Figure S1D). The virulence characteristics of the Δ*arsR6* were assessed in a murine infection model. The spleen weight decreased at a higher rate than in mice infected with the wild-type strain at 2 weeks post-infection in Δ*arsR6*-infected mice (Figure 1B). Furthermore, the bacterial load in the spleen was decreased significantly at 2 weeks post-infection in Δ*arsR6*-infected mice compared to WT strain-infected mice (Figure 1C). Spleens from mice that were infected with the WT strain exhibited a significant increase in the white-to-red pulp ratio due to white pulp expansion and macrophages’ increase at 1 and 2 weeks post-infection (Figure 1D and Figure S1E). In contrast, infection with Δ*arsR6* impacted the pathological characteristics of the spleen less severely with a modest decrease in the white-to-red pulp ratio and slight white pulp expansion at 2 weeks post-infection (Figure 1D). Altogether, these results showed that ArsR6 is required to maintain virulence in a mouse model of infection and demonstrate that ArsR6 plays an important role in *Brucella* survival both in vivo and in vitro.

### 2.2. Identification of ArsR6 Target Genes by RNA-Seq and ChIP–Seq Analyses

**Figure 2:**
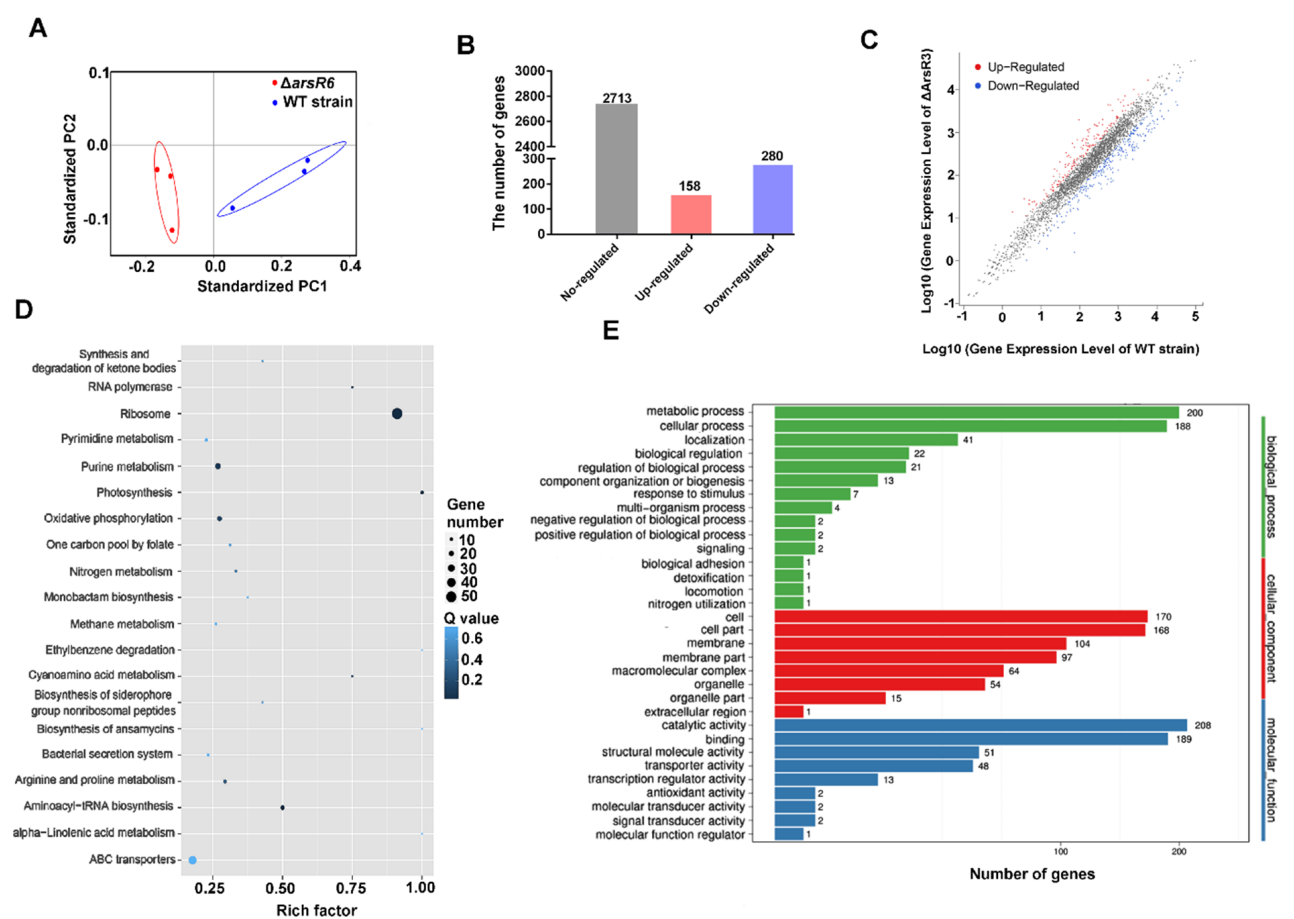
Expression of DEGs between the WT strain and ΔArsR6. (**A**) Similarities visualized among samples using MDS analysis. Blue, WT strain; red, Δ*arsR6*. (**B**) Histogram illustrating the number of DEGs and none-regulated genes between the WT and Δ*arsR6* strains. (**C**) Scatter plot of co-expressed genes between the WT and Δ*arsR6* strains. Red, blue, and gray denote upregulated, downregulated, and none-regulated genes, respectively, in Δ*arsR6* compared with the WT strain based on the following criteria: log2 (fold change) ≥ 1 and adjusted *p* value ≤ 0.05. KEGG (**D**) and GO (**E**) pathways enrich analysis of DEGs. The enrichment factor represents the ratio of DEGs annotated in this pathway term to all DEGs’ numbers annotated with this pathway term. A higher enrichment factor indicates a greater degree of pathway enrichment. The Q value represents the corrected *p* value and ranges from 0 to 1, and a lower value indicates greater pathway enrichment.

ArsR family members are involved in numerous biological processes, including metal ion homeostasis, the response to stress conditions, primary and secondary metabolism, and virulence [6,12,13,14]. Samples were evaluated using MDS analysis, which indicated that each groups’ samples had high data reproducibility (Figure 2A). The expression of 438 genes (13.9% of the total genes) was significantly different between WT and Δ*arsR6* strains, with 158 genes enhanced and 280 genes repressed by ArsR6 (Figure 2B,C). These results revealed that ArsR6 exerts a global effect on gene expression in *Brucella*. The DEGs were analyzed further using KEGG and GO pathway enrichment analysis, which demonstrated that ArsR6 is involved in assorted biological processes in *Brucella* (Figure 2D,E). Analogously, we found that many DEGs were involved in amino acid, energy, and lipid metabolism, which indicated that ArsR6 can directly or indirectly regulate diverse metabolism-related genes.

**Figure 3:**
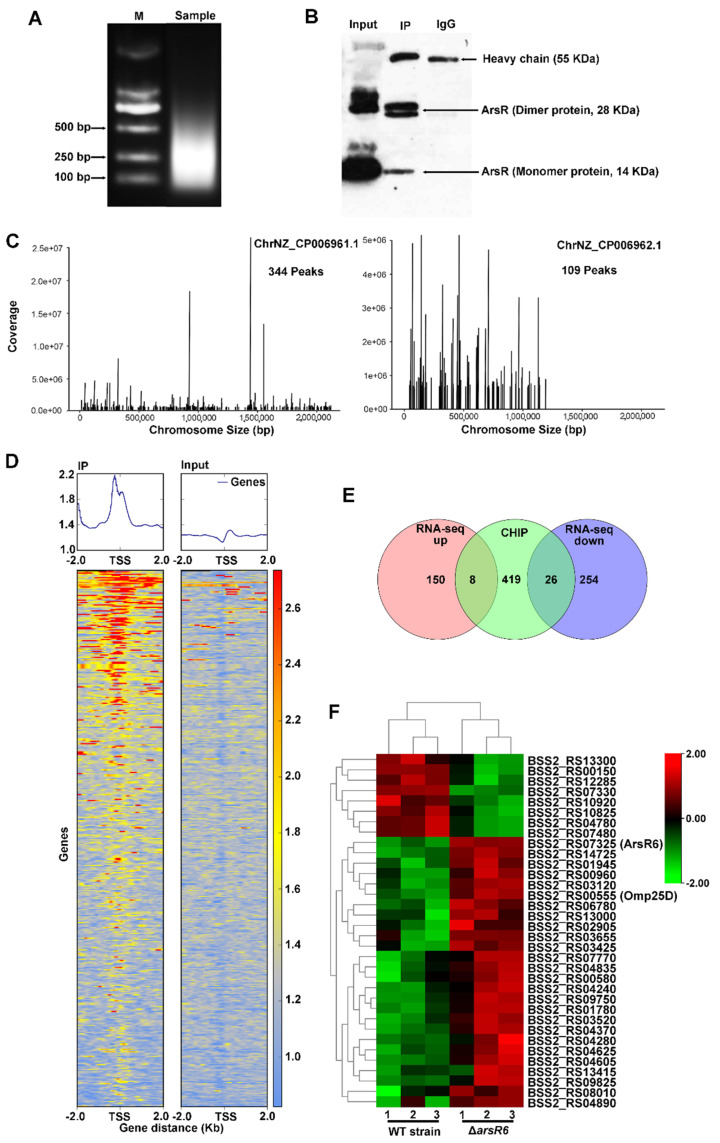
Analysis of ArsR3 target genes by ChIP-seq. (**A**) PCR identification of sample DNA fragment size after ultrasonication. M, marker. The sample was sonicated on ice by applying 10 bursts of 10 s at 50% amplitude to shear the DNA to fragments of approximately 250-bp base pairs. (**B**) Co-immunoprecipitation of ArsR6 followed by Western blot detection. Input: DNA fragment control group without antibody precipitation. IP: sample treatment group with antibody co-immunoprecipitation. IgG: antibody heavy chain control group. (**C**) ArsR6 ChIP-seq enrichment profiles in *Brucella* chromosomes. (**D**) Density plot of ArsR6 ChIP-Seq reads at 4-kb genomic regions centered at peak summits (signal intensity represents normalized tag count). (**E**) Venn diagram depicting the number of unique DEGs correlated with ArsR6 binding following nearest gene annotation. (**F**) Heat map showing the expression levels of 34 DEGs correlated with ArsR6 binding following nearest gene annotation.

As the gene expression profile of ArsR6 was revealed by RNA-seq, we next performed a genome-wide screen for ArsR6 binding sites using chromatin immunoprecipitation (ChIP). The growth conditions were chosen to match those utilized in the RNA-seq analysis. Chromatin-bound ArsR6 was crosslinked and the DNA was sheared to approximately 100–500 bp (Figure 3A). Nonspecific nucleic acids and proteins were removed and the purified DNA was analyzed by immunoprecipitation (Figure 3B). There are 344 binding sites for ArsR6 on chromosome I and 109 binding sites on chromosome II (Figure 3C). These sites were particularly enriched near to transcription start sites (Figure 3D). Examination of the Chip-seq data using KEGG and GO pathways’ enrichment analyses revealed that peak-associated genes are involved in various processes, including ABC transporters, two-component systems, metabolism, and outer membrane organization (Figure S2). Altogether, these results showed that ArsR6 has multiple binding sites on chromosome and may regulate the expression of a large number of genes.

The data from the RNA-seq and ChIP-seq were analyzed further to evaluate the genes that potentially are directly regulated by ArsR6. The set of peak-associated genes was filtered using the list of DEGs as determined by RNA-seq. We found that 34 DEGs were identified in the ChIP-seq data, among which eight genes were up-regulated and 26 genes were down-regulated, suggesting that these loci were controlled directedly by ArsR6 (Figure 3E). A heat map of the 34 DEGs was generated to visualize the DEGs (Figure 3F). The promoters of the remaining 150 up-regulated genes and 254 down-regulated genes were not bound by ArsR6, which suggested that these genes were regulated indirectly by ArsR6. Further exploration of the function of the genes that are regulated directly by ArsR6 is necessary to elucidate the effect of ArsR6 on the *Brucella* virulence.

### 2.3. Transcriptional Autoregulatory Properties of ArsR6

**Figure 4:**
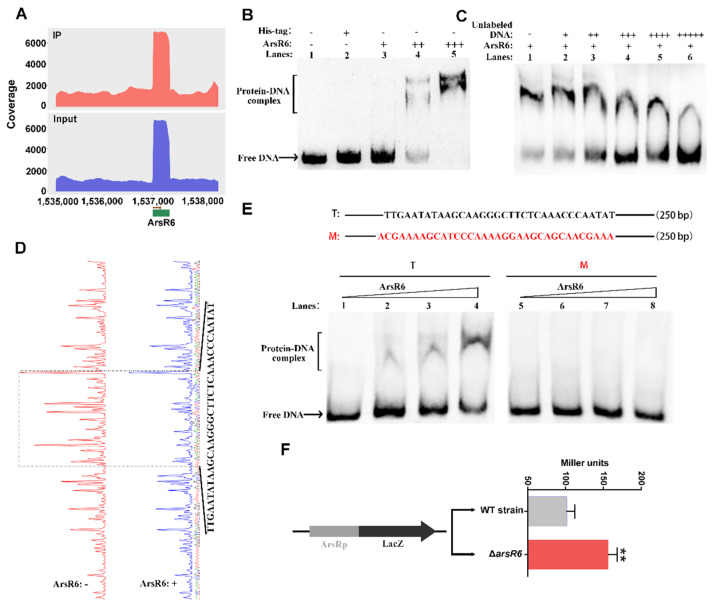
Transcriptional autoregulation of ArsR6. (**A**) The peak in the promoter region of *arsR6* by ChIP-seq analysis. The peak was generated using ArsR6 ChIP-seq data in the CΔ*arsR6* strain. (**B**) EMSA assays of ArsR6 binding to the *arsR6* promoter. Biotin-labeled substrate was incubated with free His-tag (3 μg (+)) or different concentrations of His-tagged ArsR6 (0 (-), 1 (+), 2 (++), and 3 μg (+++)). Results are representative of at least three independent experiments. (**C**) Competition assays. Unlabeled cold *arsR6* promoter sequence was tested for competition with the biotin-labeled promoter. Unlabeled cold *arsR6* promoter sequence inhibits the binding of ArsR6 to the labeled promoter. Results are representative of at least three independent experiments. -, No DNA or protein; +,++,+++,++++,+++++, add DNA or protein. (**D**) ArsR6 (2 μg) binding to the promoter region of *arsR6* by DNaseI footprinting. Nucleotide sequences protected by ArsR6 are marked by the dotted line. (**E**) EMSA for the ability of ArsR6 to bind sequences protected in DNaseI footprinting. The WT and mutated *arsR6* promoter region probes are illustrated. Probes were co-incubated with ArsR6 (0, 1, 1.5, and 2 μg). Results are representative of at least three independent experiments. (**F**) The effect of ArsR6 on expression of the cognate promoter was detected by β-galactosidase assays using an *arsR6-lacZ* transcriptional fusion. Data represent mean and standard deviation of N = 3 (independent biological replicates). The asterisks indicate significant differences (** *p* < 0.01) based on one-way ANOVA followed by Tukey’s post hoc test of honestly significant differences (two-tailed).

We found that ArsR6 was significantly enriched at the cognate promoter region in ChIP-seq (Figure 4A), which suggests that ArsR6 auto-regulates its expression. His-tagged ArsR6 and the free His-tag were purified (Figure S3A,B) and EMSA assays were performed to analyze further the interaction between ArsR6 and its promoter. During purification, we found ArsR6 forms dimers in vitro and in vivo (Figure 3B and Figure S3C). The labelled probe in EMSA comprised 250 bp upstream of the arsR6 start codon and included the promoter region. An increasing protein–DNA complex was observed with increasing concentrations of ArsR6, but not with the free His-tag (Figure 4B). In competition assay with a fixed concentration of ArsR6 and increasing concentrations of unlabeled DNA, the intensity of the protein–DNA complex gradually decreased and free DNA was gradually increased correspondingly (Figure 4C). DNaseI footprinting assays were performed to analyze further ArsR6 binding to the cognate promoter region. When increasing concentrations of ArsR6 were co-incubated with the promoter region fragment, a zone of clear protection was observed on the coding strand (Figure 4D). EMSA assays with a mutated probe were performed to assess the significance of the protected region for specific recognition by ArsR6. We observed that the intensity of the protein–DNA complex gradually increased with increasing concentrations of the protein with the wild-type promoter region, but that the complex was not observed with the mutated probe (Figure 4E). Reporter assays with an ArsR6-lacZ transcriptional fusion were conducted to determine the effect of ArsR6 on expression from the *arsR6* promoter. The *arsR6*-*lacZ* plasmid was constructed and transformed into the WT and Δ*arsR6* strains. Compared to the WT strain, the beta-galactosidase activity was increased in Δ*arsR6* (Figure 4F), indicating that ArsR6 autorepresses the activity of its cognate promoter at the transcriptional level. Taken together, our results showed that ArsR6 specifically binds the region upstream of *arsR6* to inhibit ArsR6 expression under standard conditions.

### 2.4. ArsR6 Is a Metalloregulatory Protein

**Figure 5:**
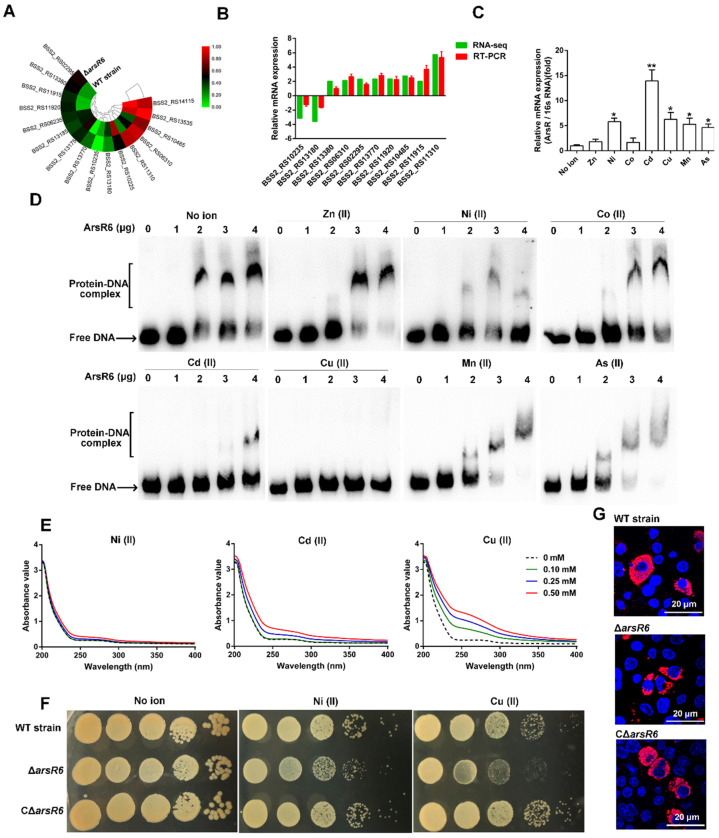
ArsR6 binds copper to maintain intracellular Cu/Ni homeostasis. (**A**) Heat map showing the expression levels of 16 DEGs correlated with metal ion-associated genes. (**B**) The expression of metal ion-associated genes was further detected by RT-PCR. Data represent mean and standard deviation of N = 3 (independent biological replicates). (**C**) The expression of *arsR6* was detected by RT-PCR with different metal ion concentrations. The WT strain was cultured with no metal supplement or with ZnSO_4_ (0.5 mM), NiSO_4_ (0.5 mM), CoSO_4_ (0.5 mM), CdCl_2_ (10 μM), CuSO_4_ (0.5 mM), and MnCl_2_ (0.5 mM). Data represent mean and standard deviation of N = 3 (independent biological replicates). * *p* < 0.05; ** *p* < 0.01 (**D**) The effect of metal ions on the DNA-binding activity of ArsR6. The *arsR6* promoter region probe was incubated with different concentrations of ArsR6 (0, 0.5, 1, 2, and 3 μg) with (0.5 mM) or without metal ions. Results are representative of at least three independent experiments. (**E**) Absorptivity spectra of ArsR6 was detected after different concentrations of Ni (II), Cd (II), and Cu (II) were added. Results are representative of at least three independent experiments. (**F**) Copper susceptibility of Brucella in vitro. Serial dilutions of log-phase cultures of the WT, Δ*arsR6*, and CΔ*arsR6* strains were spotted on TSA plates with or without CuSO_4_ (2.5 mM). Results are representative of at least three independent experiments. (**G**) Confocal microscopy analysis of the intracellular survival of the WT, Δ*arsR6*, and CΔ*arsR6* strains. Red, *Brucella*; blue, cell nucleus. Results are representative of at least three independent experiments.

We found in RNA-seq data that several DEGs, such as a putative Ni/Co exporter (BSS2_RS10485) and a Cu-processing system ATP-binding protein (BSS2_RS11310), are involved in metal ion transport (Figure 5A). To examine further the RNA-seq results, RT-PCR was performed using the mRNA libraries constructed for RNA-seq. The RT-PCR results were largely consistent with those obtained from the RNA-seq data, which confirmed the validity of the RNA-seq results (Figure 5B). Significantly, the expression of ArsR6 was enhanced significantly under several metal ion stresses (Figure 5C). 

Several studies have shown that the DNA binding activity of ArsR family members is inhibited by the presence of metal ion [7,15,16,17]. Therefore, we examined next the effect of metal ions on the binding of ArsR6 to the cognate promoter region. When Ni (II), Cd (II), or Cu (II) was co-incubated with the probe fragment that contained the *arsR6* promoter region and increasing concentrations of ArsR6, protein–DNA complex formation was disrupted (Figure 5D). However, this phenomenon was not observed with the addition of Zn (II), Co (II), Mn (II), or As (II) (Figure 5D). The results indicate that Ni (II), Cd (II), and Cu (II) specifically impair the DNA-binding ability of ArsR6 and suggest that ArsR6 may be a Ni/Cd/Cu-binding protein. We monitored ArsR6 binding to metal ion by changes in the absorption spectrum in the ultraviolet region to determine whether ArsR6 directly binds metal ion. The absorbance values were enhanced significantly with increasing concentrations of Ni (II), Cd (II), or Cu (II) (Figure 5E), but not with the addition of Zn (II), Co (II), Mn (II), or As (II) (Figure S4). Thus, the results indicated that ArsR6 is a Ni/Cd/Cu-binding protein and the metal-bound state of the protein modulates its DNA-binding properties.

To explore the relationship between ArsR6 and Cu toxicity, we assayed the copper sensitivity of ArsR6 deletion strain by spotting 10-fold serial dilutions of cultures of the same density on plates with copper. We found that the growth of Δ*arsR6* was inhibited by adding Cu (II) compared to WT strain, but fully restored in CΔ*arsR6* (Figure 5F). In addition, the same phenomenon was observed by adding Ni (II). The CCK8 assay was used to detect the cytotoxicity of Cu to RAW264.7 macrophages (Figure S5A,B). We observed that the survival of Δ*arsR6* was decreased in RAW264.7 macrophages by adding Cu (II) compared to WT strain, but fully restored in CΔ*arsR6* (Figure 5G and Figure S5C). Overall, the results demonstrate that ArsR6 is involved in maintaining Cu/Ni homeostatic levels in *Brucella*.

### 2.5. The Omp25D Gene Is a Direct Target of Transcriptional Activation by ArsR6

**Figure 6:**
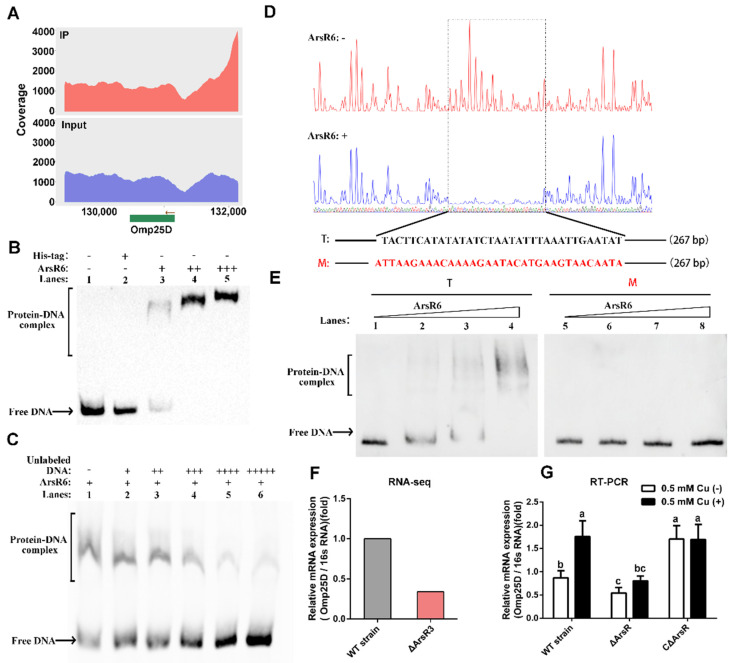
Expression of *omp25D* is regulated by ArsR6. (**A**) ArsR6 is enriched in Omp25D promoter regions in ChIP-seq analysis. (**B**) EMSA for binding of ArsR6 to the *omp25D* promoter region. Biotin-labeled substrate was incubated with free His-tagged (3 μg (+)) or different concentrations of His-tagged ArsR6 (0 (-), 1(+), 2(++), and 3 μg (+++)). Results are representative of at least three independent experiments. (**C**) Competition assays. Unlabeled cold *omp25D* promoter region was tested for competition with biotin-labeled *omp25D* promoter. Unlabeled cold *omp25D* promoter region inhibits the binding of Omp25D to the labeled promoter. Results are representative of at least three independent experiments. -, No DNA or protein; +,++,+++,++++,+++++, add DNA or protein. (**D**) Analysis of ArsR6 bound to the promoter region of *omp25D* by DNaseI footprinting. Nucleotide sequences protected by ArsR6 are marked by the dotted line. (**E**) EMSA for the ability of ArsR6 to bind nucleotide sequences protected in DNaseI footprinting. The WT and mutated *arsR6* promoter region probes are illustrated. Probes were co-incubated with ArsR6 (0, 1, 2, and 3 μg). Results are representative of at least three independent experiments. (**F**) Expression of *omp25D* in RNA-seq data. (**G**) Expression of *omp25D* was detected by RT-PCR. Data represent mean and standard deviation of N = 3 (independent biological replicates). Different small letters indicate significant differences based on one-way ANOVA followed by Tukey’s post hoc test of honestly significant differences (two-tailed).

We found that the promoter regions of several *omp* genes, including those for Omp25C, Omp25D, BSS2_RS16720, and BSS2_RS15445, were enriched in ChIP-seq analysis. (Figure 6A and Figure S6A). EMSA assays showed that ArsR6 binds the promoter regions for Omp25C, BSS2_RS16720, and BSS2_RS15445, but the expression of these genes was not significantly different in RNA-seq and RT-PCR experiments with WT, Δ*arsR6,* and CΔ*arsR6* strains (Figure S6B,C). Only *omp25D* expression was altered significantly in RNA-seq data, which suggested that this gene was a direct target for ArsR6. EMSA assays were performed to analyze further the interaction of ArsR6 with the *omp25D* promoter. A 267-bp sequence derived from the *omp25D* promoter region upstream of its start codon was employed. Protein–DNA complex formation increased gradually with increasing concentrations of ArsR6, which was not observed with the His-tag group (Figure 6B). Competition assays were performed subsequently: Using a defined concentration of ArsR6, the protein-*omp25D* promoter complex gradually decreased and free DNA gradually increased with the addition of increasing concentrations of unlabelled DNA (Figure 6C), suggesting that ArsR6 directly binds the *omp25D* promoter region. To examine further ArsR6 binding to the o*mp25D* promoter region, DNaseI footprinting assays were performed. The promoter region was clearly protected on the coding strand when increasing concentrations of ArsR6 were co-incubated with an *omp25D* fragment (Figure 6D). EMSA assays with a mutated probe were performed to assess further the binding of ArsR6 to the *omp25D* promoter region. Protein–DNA complex formation increased gradually with increasing concentrations of ArsR6 with the wild-type probe, but the mutant probe was not recognized by ArsR6 (Figure 6E). We also found that *omp25D* expression was decreased in RNA-seq data (Figure 6F), which was consistent with RT-PCR results (Figure 6G). Overall, the results showed that ArsR6 binds the *omp25D* promoter region to control *omp25D* expression.

### 2.6. Omp25D Contributes to Brucella Virulence

**Figure 7:**
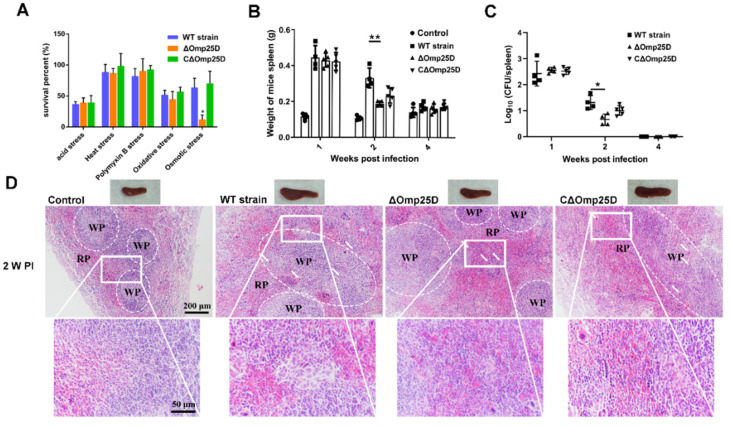
Omp25D is a *Brucella* virulence factor. (**A**) The survival of *Brucella* under osmotic stress conditions. The WT, Δ*omp25D,* and CΔ*omp25D* strains were cultivated at pH 5.5 or 50 °C or were treated with polymyxin (10 μg/mL), H_2_O_2_ (0.5 mM), or sorbitol (0.5 M). Data represent mean and standard deviation of N = 3 (independent biological replicates). The asterisks indicate significant differences (* *p* < 0.05) based on two-way ANOVA followed by Tukey’s post hoc test of honestly significant differences (two-tailed). (**B**) Spleen weights were measured at 1, 2, and 4 weeks post-infection. The control represents uninfected BALB/c female mice injected with PBS. Data represent mean and standard deviation of N = 5 (independent biological replicates). The asterisks indicate significant differences (** *p* < 0.01) based on two-way ANOVA followed by Tukey’s post hoc test of honestly significant differences (two-tailed). (**C**) The bacterial load was measured in homogenates at 1, 2, and 4 weeks post-infection. Data represent mean and standard deviation of N = 5 (independent biological replicates). The asterisks indicate significant differences (* *p* < 0.05) based on two-way ANOVA followed by Tukey’s post hoc test of honestly significant differences (two-tailed). (**D**) Representative micrographs of spleen histopathology at 2 weeks post-infection. WP, white pulp; RP, red pulp; square, magnification box.

Omps in *Brucella*, including Omp25 and Omp31, are involved in cell envelope integrity and virulence [18,19,20,21], but the significance of Omp25D is unknown. Deletion and complementary strains were constructed to assess the role of Omp25D in virulence (Figure S7A). The growth pattern of Δ*omp25D* was similar to that of the WT strain in standard medium, indicating that Omp25D did not affect *Brucella* growth in normal medium (Figure S7B). However, the survival rate of the Δ*omp25D* strain decreased under osmotic stress. This impairment was reversed fully in CΔ*omp25D* (Figure 7A). The results suggest that Omp25D may play a key role role in maintaining cell envelope integrity during osmotic shock. 

As virulence factors benefit the survival of *Brucella* in vitro and in vivo [22,23], we probed the contribution of Omp25D to pathogenicity both in RAW264.7 macrophages and in a mouse infection model. Omp25D did not affect the intracellular survival of *Brucella* in RAW264.7 macrophages (Figure S7C). However, the spleen weight was decreased at 2 weeks post-infection in Δ*omp25D*-infected mice compared to those that were infected with the WT strain (Figure 7B). The survival of Δ*omp25D* in the spleen was examined by determining the colony-forming unit (CFU) number. The bacterial load in the spleen was decreased significantly at 2 weeks post-infection in Δ*omp25D*-infected mice compared to infection with WT strain (Figure 7C). In pathological analysis, the spleens infected with the WT strain exhibited a significant increase in the white-to-red pulp ratio due to white pulp expansion and macrophage increase at 1 and 2 weeks post-infection, but this effect was fully reversed at 4 weeks post-infection (Figure 7D and Figure S7D). In contrast, infection with Δ*omp25D* reduced the pathological characteristics of the spleen with a modest decrease in the white-to-red pulp ratio and slight white pulp expansion at 2 weeks post-infection compared to spleens from mice infected with WT strain (Figure 7D). Overall, the results demonstrated that Omp25D is required to maintain bacterial virulence in a mouse model of infection. 

## 3. Discussion

The ArsR family is distributed widely among microorganisms. Although involved in diverse cellular events [15,24,25], the main function of ArsR proteins is as metal sensors that maintain intracellular homeostasis [5,26,27]. In this study, we demonstrated that ArsR6 is required for survival of *Brucella* during heat, oxidative, and osmotic stresses, as well as in a mouse infection model. RNA-seq and ChIP-seq revealed that multiple potential target genes, including *arsR6* and *omp25D*, were regulated by ArsR6. ArsR6 is a classical ArsR homologue that autoregulates its expression at the transcriptional level during standard growth conditions, but which also regulates other loci. ArsR6 disassociated from its binding sites to enhance its own expression to maintain intracellular metal ion homeostasis during heavy metal ion stress. These results are consistent with previous studies that the classical function of ArsR regulators’ response is to maintain intracellular metal ion homeostasis [6,15,25].

Although ArsR regulators are required for survival in extreme conditions, including intracellular physiological fluctuations and heavy metal ion stress [5,6,28], only a mutant of the ArsR homologue BME_RS02165 was attenuated in a mouse model of infection among four ArsR family candidates that were examined in *Brucella melitensis* [10]. These results are consistent with our findings that ArsR6 is required for *Brucella* survival during murine infection in in a mouse model of infection. Thirty-four potential target genes of ArsR6 were identified in RNA-seq and ChIP-seq analysis, which suggests that ArsR6 may be a classical ArsR family transcriptional regulator that exerts an effect on a wide array of genes. ArsR family members such as CyeR from *Corynebacterium glutamicum* and SoxR from *E. coli* respond to oxidative stress [29,30]. Analogously, our data showed that Cu-Zn superoxide dismutase SodC (BSS2_RS13300) is potential target genes for ArsR6 in *Brucella*. SodC is required for *Brucella abortus* survival during oxidative stress, as well as the establishment and maintenance of persistent infection [31]. These results correlated with the decreased survival rate of the ArsR6 mutant of *Brucella* here during oxidative stress. We also found that *omp25D* is a direct target gene of ArsR6 and that deletion of *omp25D* attenuated *Brucella* in a mouse model of infection. Thus, ArsR6 directly regulates expression of the Omp25D virulence factor to benefit *Brucella* survival. *Brucella* Omps play important roles in the maintenance of cell envelope physiological function [32]. The decreased survival rate of the Omp25D deletion strain may reflect the impairment of cell membrane physiological function. The combination of the effects of ArsR6 on diverse loci may explain attenuation of the ArsR6 deletion mutant in mice, although the molecular mechanism by which the ArsR6 deletion strain is defective remains unclear. Nevertheless, it is apparent that ArsR6 is crucial to escape host killing during persistent infection by *Brucella*.

ArsR family members autoregulate their expression by binding to the cognate promoters in standard growth conditions or at low concentration of metals but disassociate from the binding sites to increase their own expression to sequester excess heavy metal ions in extreme conditions [5,6]. In this study, we found that ArsR6 possesses self-inhibitory properties similar to numerous ArsR family members [5,17,33]. The DEGs in RNA-seq data were associated with metal ion transport, such as a Ni/Co exporter (BSS2_RS10485) and a Cu-processing system ATP-binding protein (BSS2_RS11310), suggesting that ArsR6 is a metalloregulatory protein. Accordingly, we found that ArsR6 is a metal sensor. Low concentrations of Cu are important as a cofactor of cytochrome c oxidase and Cu-Zn superoxide dismutase during bacterial growth, but excess Cu is toxic for many bacteria and the host capitalizes on this toxicity to defend against bacterial invaders [8,9,34]. Growth of the ArsR6 deletion strain was no different from the WT strain in standard conditions in vitro but was inhibited by excess Cu compared to the parental strain. This phenomenon also was observed in RAW264.7 macrophages. These findings show that ArsR6 is a classical ArsR family member that is involved in maintaining cellular Cu homeostasis, which may partly explain why the ArsR6 deletion strain is attenuated in mice.

As important components of the cell wall, Omps are highly conserved in *Brucella* and are required for virulence, including Omp10, Omp19, Omp22, Omp25, and Omp31 [19,35,36]. The deletion of the gene for Omp25D from rough *Brucella ovis* PA led to a significant reduction in invasion and survival inside host cells as well as in spleen colonization in mice [19,37], although no evidence supported a role for Omp25D in virulence in smooth strains. In this study, we found that the absence of Omp25D did not affect bacterial survival in standard conditions in vitro or in RAW 264.7 macrophages in vivo. This may reflect a compensatory mechanism within group 3 Omps such that in the absence of Omp25C, Omp25D and Omp3B increase Omp25B production [38]. A balance of the group 3 Omps seems to be important for the integrity and physiological function of the cell envelope [37]. However, we found that Δ*omp25D* is attenuated in mice, which suggests that the increase of Omp25B is not sufficient to compensate for the absence of Omp25D during infection. Thus, we revealed a novel regulatory mechanism in which ArsR6 regulates Omp25D production to maintain the physiological status of the cell envelope. 

In conclusion, our results revealed that ArsR6 is a novel virulence factor in *Brucella*. ArsR6 plays a critical role in avoiding host killing by autoregulating its expression to maintain metal ion homeostasis and by enhancing Omp25D production to maintain the physiological function of cell membranes. Our findings help expand the understanding of the mechanisms by which bacterial pathogens escape host killing during the process of infection.

## 4. Materials and Methods

### 4.1. Ethics Statement

All animal experiments were conducted in accordance with the “Guidelines on Ethical Treatment of Experimental Animals” (2006) No. 398 from the Ministry of Science and Technology, China. The sampling procedures used in the present study received prior approval from the Experimental Animal Manage Committee of Northwest A&F University with the approval license number 2018ZX04002032.

### 4.2. Bacterial Strains and Growth Conditions

*Escherichia coli* DH5α and BL21 (DE3) (TaKaRa, Dalian, China) were grown in liquid Luria-Bertani (LB) medium at 37 °C for genetic manipulations and protein expression, respectively. *Brucella suis* S2 (WT strain) (CVCC reference number CVCC70502) was obtained from the Shaanxi Provincial Institute for Veterinary Drug Control (Xi’an, Shaanxi, China). Antibiotics were used at the following concentrations (μg/mL) when required: kanamycin, 50; ampicillin, 50; and gentamicin, 25 or 50. The *arsR6* and *omp25D* gene deletion strains (Δ*arsR6* and Δ*omp25D*, respectively) were acquired by allelic exchange, as described previously [39]. The Δ*arsR6* and Δ*omp25D* complementing strains (CΔ*arsR6* and CΔ*omp25D*) were acquired, as described previously [39]. All specific primers are listed in Supplementary Table S1.

### 4.3. Stress Resistance Assays

The WT strain and its derivatives were cultured in tryptone soybean broth (TSB, Sigma, St. Louis, MO, USA) at 37 °C with shaking to an optical density at 600 nm (OD_600_) of ~0.6 and the number of bacterial colony-forming unit (CFUs) was determined by 10-fold gradient dilution. The 10^5^ CFUs of the WT strain and its derivatives were treated in 1 mL TSB for 1 h under the following conditions: pH 5.5, water bath at 42 °C, 10 μg/mL polymyxin B, 0.5 Mm H_2_O_2_, and 0.5 M sorbitol. After being treated, the survival rate was confirmed by 10-fold gradient dilution in PBS and plated onto tryptone soybean agar (TSA, Sigma, St. Louis, MO, USA) for 72 h to determine.

### 4.4. Macrophage Infection

RAW264.7 macrophages (National Collection of Authenticated Cell Cultures) were cultured at Roswell Park Memorial Institute (RPMI) 1640 (hyclone laboratories Inc., Logan, UT, USA), supplemented with 10% fetal bovine serum (FBS; Gibco) at 37 °C with 5% CO_2_. Briefly, RAW264.7 macrophages were seeded in 24-well plates at a density of 2 × 10^5^ cells/well. Cells were infected with bacteria after 12 h at a multiplicity of infection of 100:1. After 1 h, the cells were washed three times and further incubated for 1 h with cell culture medium containing gentamicin (50 µg/mL) to eliminate extracellular bacteria. Cells then were cultured in cell culture medium containing gentamicin (25 µg/mL) to avoid continuous infection. For CFU counting, RAW264.7 macrophages were washed three times with PBS and lysed in PBS containing 0.5% Triton X-100 (0.5 mL) at each time point. The lysates were diluted serially 10-fold and then cultured on TSA plates for 72 h at 37 °C. The number of CFUs was determined. For immunofluorescence assays, RAW264.7 cells infected with the WT strain and its derivatives were washed three times with PBS and fixed with paraformaldehyde (4%) for 30 min at room temperature. Cells then were incubated with PBS containing Triton X-100 (0.25%) at room temperature for 20 min and washed three times with PBS. Goat anti-brucella polyclonal antibody (1:100 dilution, Noncommercial antibody) and donkey anti-goat Alexa Fluor 555 antibody (1:500 dilution, Invitrogen Inc., Carlsbad, CA, USA) were used as the primary and secondary antibodies, respectively. Subsequently, coverslips were mounted onto the glass slides and the cells were observed under a microscope (Nikon, Tokyo, Japan).

### 4.5. Real-Time PCR Analysis

The WT strain and its derivatives were cultured in TSB at 37 °C with shaking to an OD_600_ of ~0.6 and were collected by centrifuge at 5000 rpm for 5 min. Total RNA was extracted with TRIzol (Invitrogen Inc., Carlsbad, CA, USA). Reverse transcription of RNA was performed using a RT Reagent kit (Vazyme Biotech Co., Ltd., Nanjing, China), and RT-PCR was conducted using an SYBR Premix Ex Taq™ (Vazyme Biotech Co., Ltd., Nanjing, China) on the ABI 7500 system (Applied Biosystems, Waltham, MA, USA). In addition, no-template control and no-reverse transcriptase control were performed in RT-PCR. The analysis of the relative transcription levels used the 2^−ΔΔCt^ method. Gene quantitative expression was normalized to the 16S ribosomal RNA gene for *Brucella*. All RT-PCR primers are listed in Supplementary Table S1.

### 4.6. Mouse Infection

All experiments were performed with 6- to 8-week-old BALB/c female mice (Experimental Animal Center, Xi’an Jiaotong University, Shaanxi, China). The mice were infected intraperitoneally with a total dose of approximately 10^7^ CFUs of the WT strain and its derivatives in PBS (200 μL). Groups of five mice per strain were sacrificed by cervical dislocation at 1, 2, and 4 weeks post infections. Spleen tissues were weighed to evaluate splenomegaly. Bacterial loads in the mice spleen were counted through homogenization in PBS, plating of serial dilutions on TSA plates, and growth at 37 °C for 72 h. At the same time, spleens were stained by hematoxylin-eosin (H & E) staining kit (Solarbio Life Science, Beijing, China) to evaluate the pathological feature, according to the manufacturer’s protocols.

### 4.7. RNA-Sequencing Analysis

Total RNA was sequenced using the Illumina Hiseq 2500 sequencer (Illumina company, San Diego, CA, USA). The reference genome data were downloaded from the NCBI database (NZ_CP006961/NZ_CP006962). Clean reads (remove low-quality reads (Q20 < 20), reads containing poly-N sequences, and adaptor sequences by FASTX-Toolkit) were aligned to the bacterial reference genome using HISAT40 software [40]. Principal component analysis (PCA) of RNA-seq data was performed by R package ade4 [41]. The relative transcript abundance was calculated as fragments in reads per kilobase of exon sequence per million mapped sequence reads (FPKM) using RESM software [42]. Only genes that demonstrated the adjusted *p* value ≤ 0.05 and the absolute value of log2 ratio ≥ 1 were identified as differentially expressed genes (DEGs). The DEGs’ Volcano plot was drawn by R package ggplot2 [41]. DEGs were subjected to Kyoto Encyclopedia of Genes and Genomes (KEGG) and Gene Ontology (GO) Pathway enrichment analysis using the online gene function analysis website DAVID (https://david.ncifcrf.gov/ (accessed on 13 September 2020)). All RNA-seq data were uploaded to Dryad (https://doi.org/10.5061/dryad.kkwh70s56 (accessed on 8 July 2021)).

### 4.8. Chromatin Immunoprecipitation Sequencing Analysis

Exponentially growing CΔ*arsR6* (OD_600_~0.6) were collected by centrifugation and were fixed with sodium phosphate buffer (10 mM; pH 7.6) and formaldehyde (1%) for 10 min at room temperature followed by 30 min on ice. CΔ*arsR6* were collected and resuspended in lysis buffer (1.1% TritonX-100, 1.2 mM EDTA, 16.7 mM Tris-HCl pH 8.0, 167 mM NaCl, protease inhibitors). The lysate was sonicated on ice by applying 10 bursts of 10 s at 50% amplitude to shear the DNA to fragments of approximately 250 base pairs. The fragments were immunoprecipitated with anti-flag mouse monoclonal antibody (TransGen Biotech, Beijing, China). Reverse crosslinking was conducted in 200 mM NaCl solution at 65 °C for 12 h and DNA was extracted and resuspended in elution buffer (1% SDS and 0.1M NaHCO_3_). ChIP DNA was assayed using Illumina HiSeq sequenator. All library preparation and sequencing were performed using standard Illumina protocols. Sequence reads were mapped to the *B. suis* S2 genome using MAQ [43]. Peak finding was performed using MACS14 [44]. The DEGs’ Venn diagram was prepared using Microsoft PowerPoint 2013. Cis-regulatory Element Annotation System (CEAS) was used to measure the average ChIP enrichment signals in a region of ±2 kbp from the TSS of genes [45]. The DEGs’ heatmap was drawn by R package ggplot2 [41]. All ChIP-seq data were uploaded to Dryad (https://doi.org/10.5061/dryad (accessed on 8 July 2021)).

### 4.9. Protein Expression and Purification

ArsR6 protein was purified, as described previously [46]. The *arsR6* coding region was amplified by PCR from WT strain genomic DNA using primers ArsR6 (ORF)-F and ArsR6 (ORF)-R, and was cloned into the prokaryotic expression vector pET-32a using the ClonExpress II One Step Cloning Kit (Vazyme Biotech Co., Ltd, Nanjing, China). Empty plasmid pET-32a and recombinant vector pET-32a-ArsR6 were transformed separately into *E. coli* BL21 (DE3). *E. coli* BL21 (DE3) carrying the relevant plasmid was cultured until OD_600_ = 0.6, and isopropyl β-D-1-thiogalactopyranoside (0.5 mM) was added to induce protein expression. Expression continued at 37 °C for 15 h. Subsequently, the induced culture was collected by centrifugation, resuspended in PBS, and lysed by sonication. Finally, the supernatant was collected by centrifugation at 12,000 rpm for 30 min. Purification of His-tagged ArsR6 and the free His-tag was performed with BeyoGold™ His-tag Purification Resin (Beyotime, Shanghai, China) according to the manufacturer’s recommended protocols. All primers are listed in Supplementary Table S1.

### 4.10. Electrophoretic Mobility Shift Assay

DNA probes were amplified by PCR using primers listed in Supplementary Table S1. The probes were labeled by biotin using an EMSA probe biotin labeling kit (Beyotime, Shanghai, China), according to the manufacturer’s recommended protocols. The labeled probe was mixed with increasing concentrations of protein in gel-shift buffer (10 μL). After incubation at room temperature for 20 min, the samples were analyzed by polyacrylamide gel electrophoresis (5%) at 80 V for 40 min. DNA was detected using the chemiluminescent biotin-labeled nucleic acid detection kit (Beyotime, Shanghai, China) according to the manufacturer’s recommended protocols. The gel was scanned by a gel imaging system (Bio-Rad, Hercules, CA, USA).

### 4.11. DNaseI Footprinting Assay

For preparation of fluorescent FAM-labeled probes, the ArsR6 and Omp25D promoter regions were PCR amplified using primers ArsR6p-F and ArsR6p-R, and Omp25Dp-F and Omp25Dp-R, respectively. The probes were labeled with FAM and purified by the Wizard^®^ SV Gel and PCR Clean-Up System (Promega, Madison, WI, USA) and were quantified with a NanoDrop 2000C (Thermo, Waltham, MA, USA). DNase I footprinting assays were performed. For each assay, probes (250 ng) were incubated with increasing concentrations of ArsR6 protein in a total volume of 40 µL. After incubation for 30 min at 25 °C, DNase I (approximately 0.015 units in 10 μL; Promega, Madison, WI, USA) and freshly prepared CaCl_2_ (100 nmol) were added and incubation was continued at 37 °C for 1 min. The reaction was stopped by adding DNase I stop solution (140 µL of 200 mM unbuffered sodium acetate, 30 mM EDTA, and 0.15% SDS). Samples were extracted, firstly, with phenol/chloroform and then precipitated with ethanol. Pellets were dissolved in MiniQ water (30 µL). The GeneScan-LIZ600 size standard (Applied Biosystems, Waltham, MA, USA) was used to assess fragment sizes.

### 4.12. Western Blot

The bacterial lysate was generated using 5 × SDS-PAGE loading buffer. Proteins were separated via 15% SDS-PAGE in Tris/glycine buffer and then transferred onto PVDF membranes. The membranes were blocked with Tween-20 (0.5%) with 5–10% nonfat dry skim milk powder for 2 h at room temperature. Membranes then were incubated with the primary Flag antibody (Zhongshan Golden Bridge Biotechnology, Nanjing, China) overnight at 4 °C and then with the HRP-conjugated goat anti-mouse secondary antibody (Zhongshan Golden Bridge Biotechnology, Nanjing, China). The blots were visualized using the Gel Image System (Tannon, Biotech, Shanghai, China).

### 4.13. Cell Counting Kit-8 (CCK8) Assay

RAW264.7 macrophages were cultured in a 96-well plate at a density of 1000 cells/well. After Cu treatment for 24 h, CCK-8 was added according to the manufacturer’s recommended protocols (Dojindo, Kumamoto, Japan). Each well absorbance was read at 450 nm with a fully automatic microplate reader (SpectraMAX 190, Silicon Valley, CA, USA).

### 4.14. Ultraviolet Light Analysis

The ArsR6 protein (0.5 mg/mL; 1.5 mL) was placed in a quartz cuvette (10 mm). Different concentrations of Cu (II), Ni (II), Co (II), and Mn (II) were added. The absorptivity spectra were detected using a spectrophotometer (Hitachi Limited, Tokyo, Japan).

### 4.15. β-Galactosidase Activity Assay

Three fragments were amplified by PCR using the primers pBBR1-F/pBBR1-R, A^+^ (LacZ)-F/A^+^ (LacZ)-R ArsR6p (LacZ)-F/ArsR6p (LacZ)-R, and LacZ-F/LacZ-R and linked using the ClonExpress II One Step Cloning Kit (Vazyme Biotech Co., Ltd., Nanjing, China) to construct the pBBR1-ArsRp-LacZ plasmid. This reporter plasmid was transformed into the WT, ΔArsR, and CΔArsR strains. All strains were grown at 37 °C to an OD_600_ = 0.6. Bacterial suspensions containing equivalent numbers of cells were collected and washed twice with cold PBS. Then, 600 μL of Z buffer (60 mM Na_2_HPO_4_, 40 mM NaH_2_PO_4_, 10 mM KCl, 1 mM MgSO_4_, 59 mM β-mercaptoethanol, pH 7.5) were added to permeabilize bacteria; 300 μL of resuspended cells were saved to determine the A_600_ values. SDS (0.1%) and chloroform (100 μL) were added, and the samples were vortexed and incubated at 28 °C for 5 min. O-nitrophenyl-β-D-galactoside (200 μL of 4 mg/mL) was then added and samples were incubated at room temperature for 5 min. Then, 1 M Na_2_CO_3_ (500 μL) was added to end the reactions. Samples were centrifuged and the supernatants were collected to determine OD_420_, OD_550,_ and OD_595_ values. Miller units were calculated using the formula: Miller units = 1000 × (OD_420_ − 1.75 × OD_550_)/T (min) × V (mL) × OD_600_.

### 4.16. Statistical Analysis

Statistical comparisons were performed using SPSS version 22 software. Graphpad prime 8.0 software was used for drawing the map. The results were presented as the means ± standard deviations (SDs). Further analyses were performed using unpaired, two-tailed Student’s t test and one-way or two-way analysis of variance (ANOVA) followed by Tukey’s post hoc test of honestly significant differences (two-tailed). Probability (*p*) values < 0.05 were considered statistically significant.

## Supplementary Materials

The Supplementary Materials are available online at https://www.mdpi.com/article/10.3390/ijms221910860/s1.
