# Supplementary material for: An ArsR Transcriptional Regulator Facilitates Brucella sp. Survival via Regulating Self and Outer Membrane Protein
Source: Int J Mol Sci. 2021 Oct 8;22(19):10860. doi: 10.3390/ijms221910860 (PMC8509827; doi:10.3390/ijms221910860)
Supplement: Supplementary file 1 [file ijms-22-10860-s001.zip › ijms-1386671-supplementary.pdf]

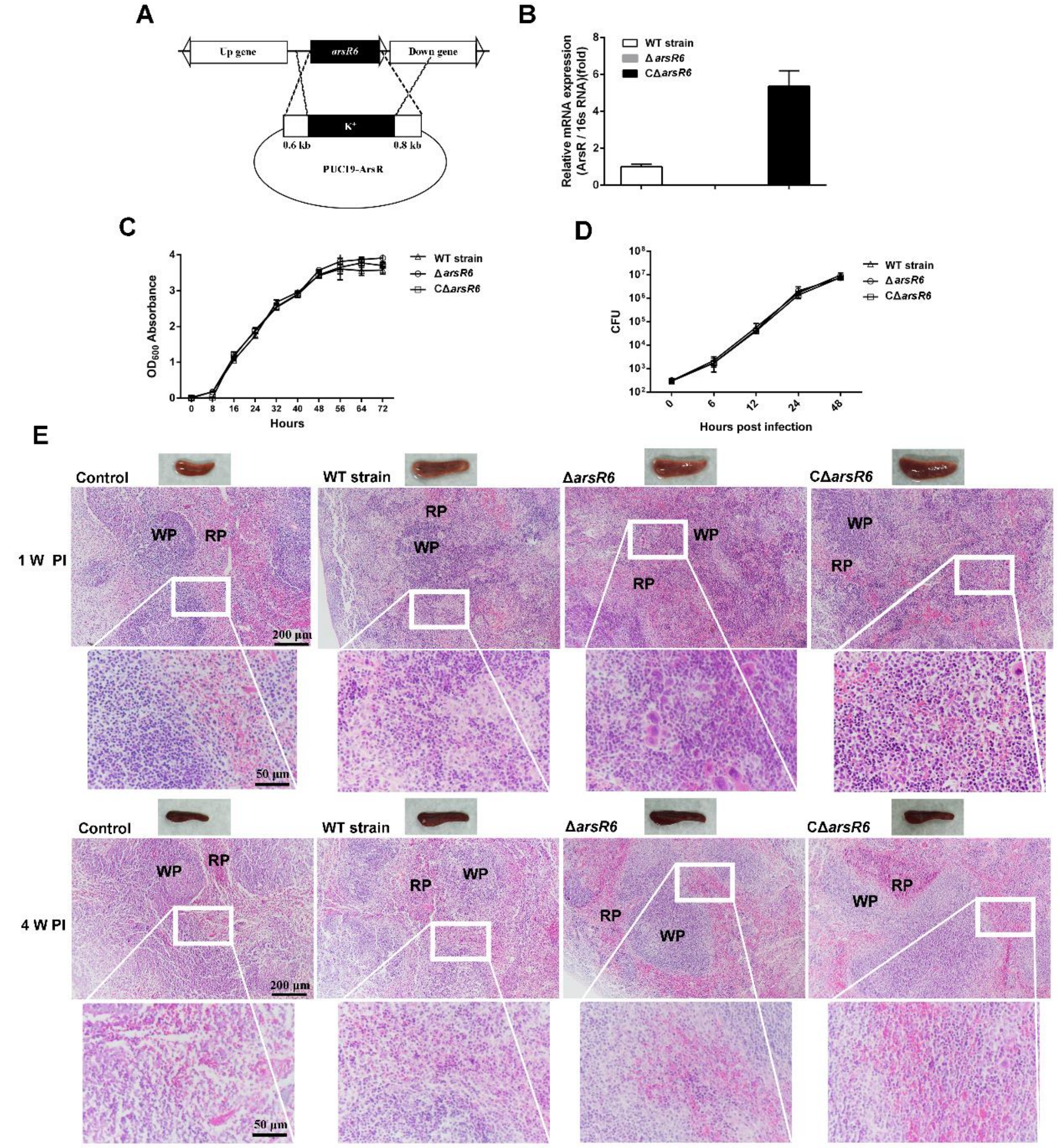

Fig. S1 Phenotypic characterization of Δ*arsR6*. (A) Map of the construction of the ArsR6 deletion strain Δ*arsR6*. **(B)** The ArsR6 deletion strain and complemented strain were assessed by RT-PCR. Data represent mean and standard deviation of N = 3 (independent biological replicates). **(C)** Growth was measured in standard conditions. Data represent mean and standard deviation of N = 3 (independent biological replicates). **(D)** Bacterial intracellular survival was assayed during *Brucella* infection in RAW264.7 macrophages. Data represent mean and standard deviation of N = 3 (independent

biological replicates). **e** Representative micrographs of the spleen histopathology at one and four weeks post-infection.

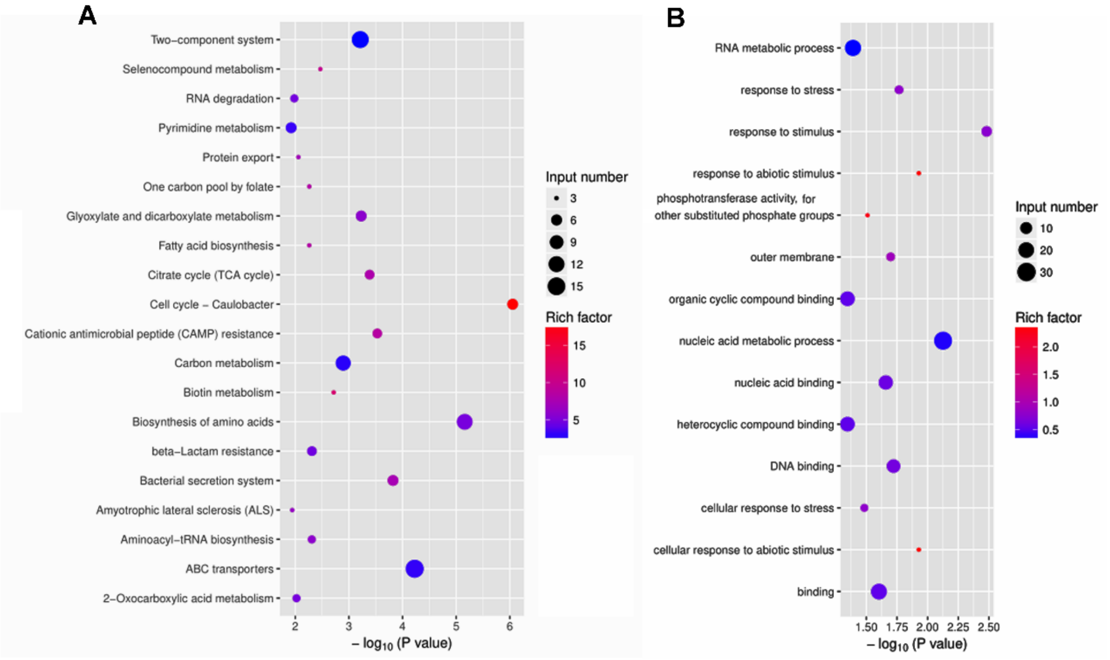

Fig. S2 KEGG (A) and GO (B) pathway enrichment analysis of peak genes in ChIP-seq. The enrichment factor represents the ratio of peak genes annotated in this pathway term to all peak genes numbers annotated with this pathway term. A higher enrichment factor indicates a greater degree of pathway enrichment. The Q value represents the corrected *P* value and ranges from 0 to 1, and a lower value indicates greater pathway enrichment.

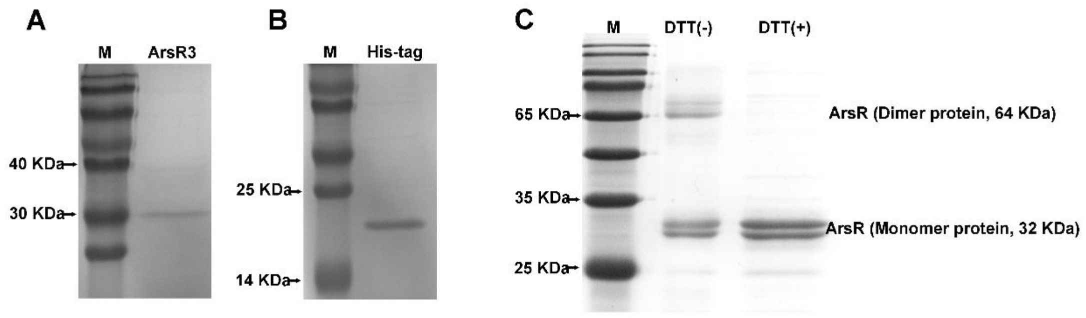

Fig. S3 The purification of His-tagged ArsR6 and His-tag. (A) SDS-PAGE of the purified His-tagged ArsR6. M, marker; ArsR6, purified recombinant ArsR6. (**B)** SDS-PAGE of the purified His-tag. M, marker; His-tag: purified His-tag. **(C)** ArsR6 forms dimers *in vitro*. ArsR6 was treated with (10 mM) or without dithiothreitol in PBS as indicated on the top. Native-PAGE of the treated sample was conducted. M, marker.

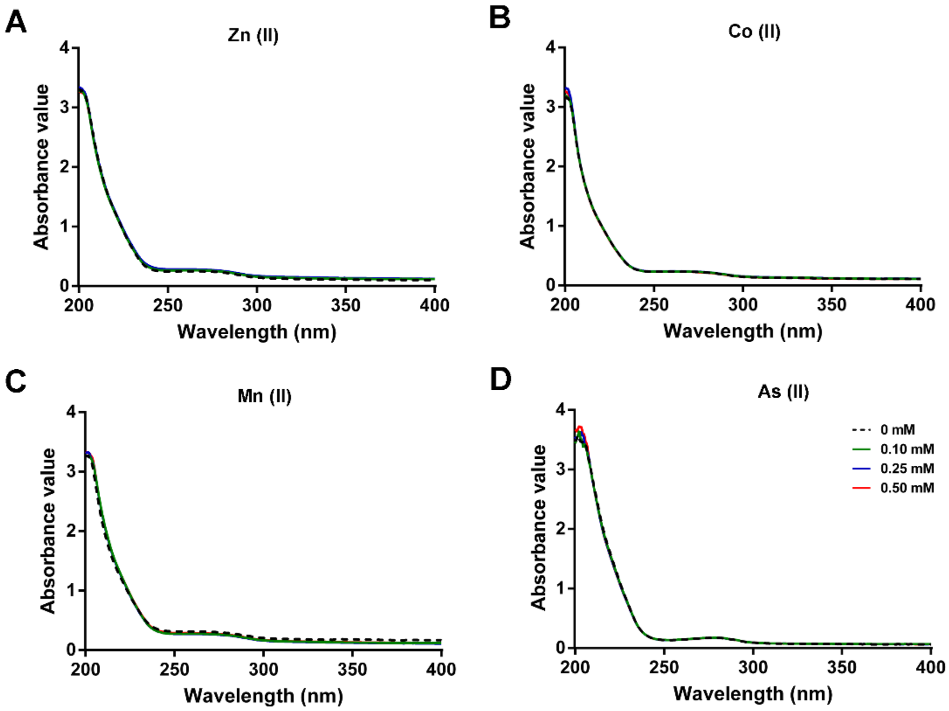

Fig. S4 Absorptivity spectra of ArsR6 were acquired after different concentrations were added. Results are representative of at least three independent experiments.

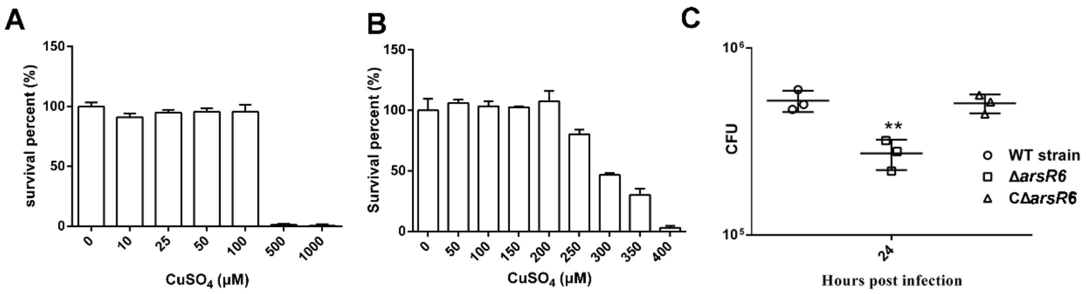

Fig. S5 Effect of Cu on the intracellular survival of Δ*arsR6*. (A) RAW264.7 macrophage viability assay after Cu treatment for 24 h. Data represent mean and standard deviation of N = 3 (independent biological replicates). **(B)** RAW264.7 macrophage viability assay after Cu treatment for 24 h. Data represent mean and standard deviation of N = 3 (independent biological replicates). **(C)** Copper susceptibility in RAW264.7 macrophages. The bacterial survival was detected by CFU at 24 h post infection after $CuSO_4$ (200 μM) was added. Data represent mean and standard deviation of N = 3 (independent biological replicates). The asterisks indicate significant differences (**$P < 0.01$) based on one-way ANOVA followed by Tukey's post-hoc test of honestly significant differences (two-tailed).

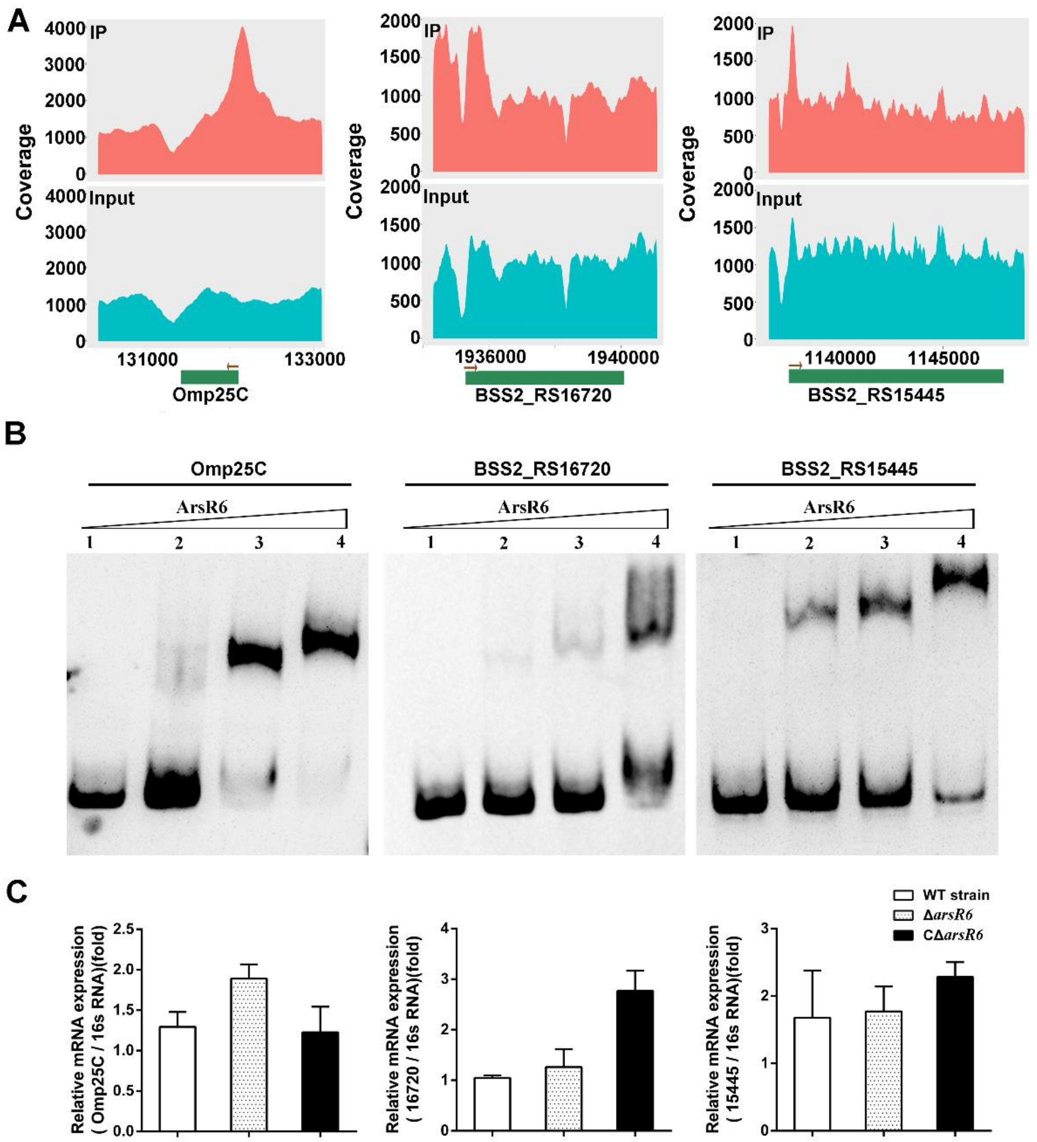

Fig. S6 Three membrane proteins were not regulated by ArsR6. (A) ArsR6 is enriched in Omp25C, BSS2_RS16720 and BSS2_RS15445 promoter regions in ChIP-seq analysis. **(B)** EMSA for the binding of ArsR6 to the promoter regions of the genes for Omp25C, BSS2_RS16720 and BSS2_RS15445. Biotin-labeled DNA substrate was incubated with different concentrations of His-tagged ArsR6 (0, 1, 2 and 3 μg). Results are representative of at least three independent experiments. **(C)** Expression of o*mp25C*, BSS2_RS16720 and BSS2_RS15445 was detected by RT-PCR. Data represent mean and standard deviation of N = 3 (independent biological replicates).

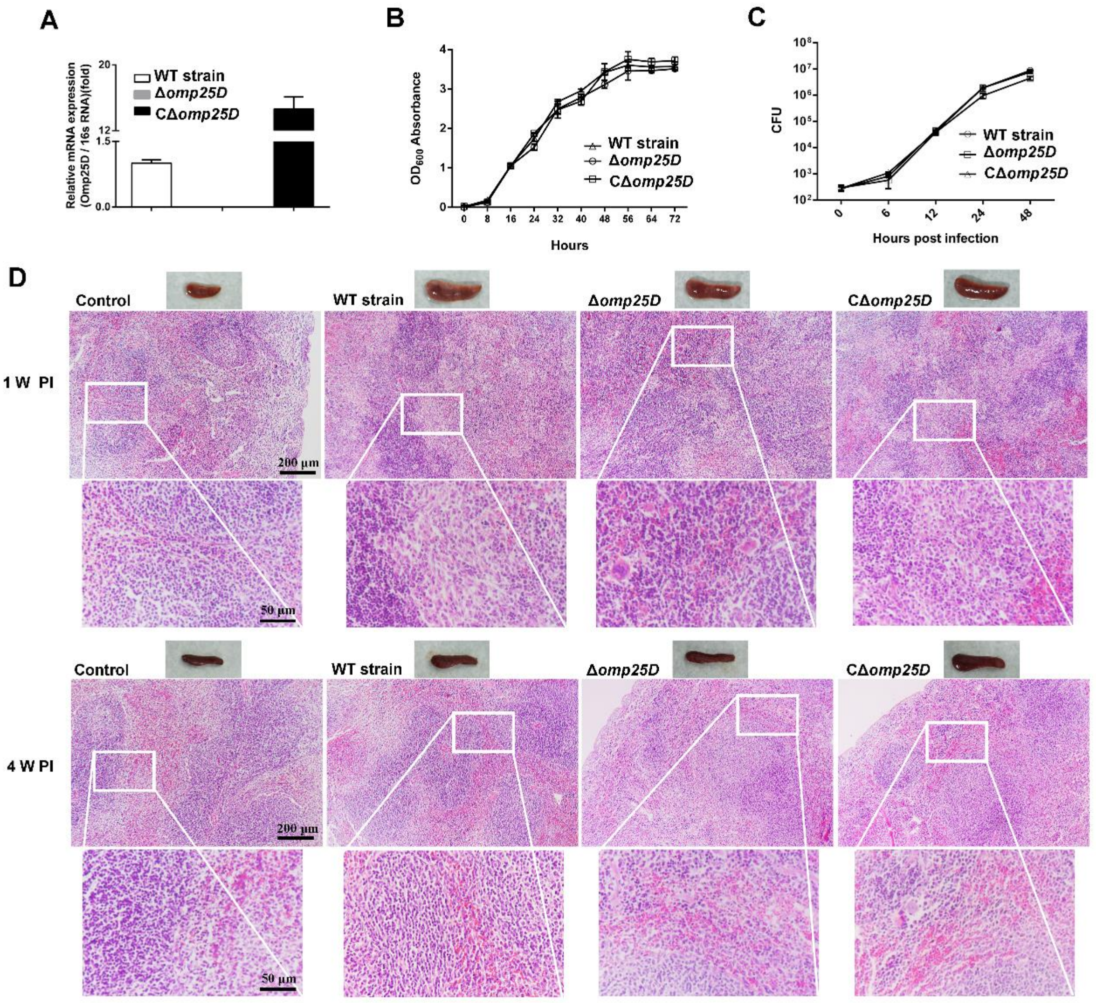

Fig. S7 Phenotypic characterization of the Δ*omp25D* strain. (A) The *omp25D* deletion strain and the complemented strain were confirmed by RT-PCR. Data represent mean and standard deviation of N = 3 (independent biological replicates). **(B)** Growth was measured in standard conditions. Data represent mean and standard deviation of N = 3 (independent biological replicates). **(C)** Bacterial intracellular survival was assayed during *Brucella* infection in RAW264.7 macrophages. Data represent mean and standard deviation of N = 3 (independent biological replicates). **(10)** Representative micrographs of the spleen histopathology at one and four weeks post-infection.

Table S1 All primers were listed for constructing the deletion and complementary strain

Gene	Forward primer (5'-3')	Reverse promer (5'-3')	Length (bp)
Upstream (ArsR6)	CTCGGAAACTTTTGCCTTTTCCATC	CAAGAACTCTGTAGCACCGCTTGCCGTCATACCCCCGTAAG	573
K^+(ArsR6)	TTACGGGGGTATGACGGCAAGCGGTGCTACAGAGTTCTTGA	GCGGCCTCATGGGATGTGGTCAGGTGGCACTTTTCGGGGA	1269
Downstream (ArsR6)	TCCCCGAAAAGTGCCACCTGACCACATCCCATGAGGCC	CAAGAGCGGCGTTGATGT	647
ArsR6 (RT-PCR)	GATCTCAGCCAGTCTGCACT	CATATAGAGGCCGGAGAGCG	150
ArsR (C)	TTTTATCAGGCTCTGGGAGGTGGCCAGGCTAACGTTTGC	AAACAAATAGGGGTTCCGCGTTACTTATCGTCGTCATCCTTGTAATCTTGGTGCGCCACCGC	662
A^+ (ArsR-C)	AGGATGACGACGATAAGTAACGCGGAACCCCTATTTGTTTATTTT	GGCGGTCACGACTTTGCGAATTACCAATGCTTAATCAGTGAGGCA	1006
pBBR1 (C)	TTCGCAAAGTCGTGACCGCCTA	CCTCCCAGAGCCTGATAAAAACG	1741
upstream (Omp25D)	ACCTTTGAAAACCGCCTTCG	CAAGAACTCTGTAGCACCGCTGAAAACTCCTAATAAGAATGGCGT	620
K^+(Omp25D)	ATTCTTATTAGGAGTTTTCAGCGGTGCTACAGAGTTCTTGA	CCGGTTCTGTGCGTCGGCAACAGGTGGCACTTTTCGGGGA	1269
Downstream (Omp25D)	TCCCCGAAAAGTGCCACCTGTTGCCGACGCACAGAACC	GTGCGGTCAGGCGAAAGA	820
Omp25D (RT-PCR)	TGACCGTTTCATGCCGTACA	TAATCGATGCCTGCTCCGAC	132
Omp25D (C)	TCAGGCTCTGGGAGGTAGTTTGGTTGAAATCTACGAAAAG	AATAGGGGTTCCGCGTTACTTATCGTCGTCATCCTTGTAATCGAACTTATAGGCAACGC	1014
A+ (Omp25D-C)	AGGATGACGACGATAAGTAACGCGGAACCCCTATTTGT	GGCGGTCACGACTTTGCGAATTACCAATGCTTAATCAGTGAGGCA	1004

BSS2_RS10235 (RT-PCR)	CCTTATTTCACATGCCGCCG	TGGATATGCAGATGGTCGCC	207
BSS2_RS13180 (RT-PCR)	TGATCCAGACGCTCGTTGTC	AATAAAGCGTGCCTGCGAAC	117
BSS2_RS13380 (RT-PCR)	ACGAACGTCCGAAGACGAAA	AGACGGCTTCCTGCATTTCA	95
BSS2_RS06310 (RT-PCR)	CATGGTCGGGCTATTGTTGC	GGAAAAGCCAGCCGAAAACA	113
BSS2_RS02295 (RT-PCR)	GTCGGTATCGGCTTCATGGT	GCAAGGATTGCAGCTTCGAG	122
BSS2_RS13770 (RT-PCR)	GTCATTACCCTGGCGCTGAT	CCGAGCATACCGACCATGAA	158
BSS2_RS11920 (RT-PCR)	GCCGCAATTCATTCCTCGAC	AGCGTTTCGAGCATGGAAGA	262
BSS2_RS10485 (RT-PCR)	TGTCGACCCATCGACACTTG	CGTGTTCTTTGCGGTTACGG	223
BSS2_RS11915 (RT-PCR)	TCCTGATCGGTGCGATTTCC	GAAAGCACGGGCCATGAAAG	181
BSS2_RS11310 (RT-PCR)	AAAAGCGCCATTTGCAGACC	CTTATGGCGTTAAAGCCGCC	103
ArsR6 (ORF)	GCGGCCGCAAGCTTGTCGACATGACTAACAAAGTTACTTTTTATG	CTGATATCGGATCCGAATTCTTGGTGCGCCACCGCCAT	385
ArsR6p	TGGCCAGGCTAACGTTTGCA	TTGCCGTCATACCCCCGT	250
Omp25Dp	TGAAAACTCCTAATAAGAATGGCGT	TAGTTTGGTTGAAATCTACGAAAAG	267
ArsR6p (LacZ)	AAACAAATAGGGGTTCCGCGTGGCCAGGCTAACGTTTGCA	TCACGACGTTGTAAAACGACTTGCCGTCATACCCCCGT	290

A^+	TTTTATCAGGCTCTGGGAGGTTACCAATGCTTAATCAGTGAGGCA	CTTGATGCAAACGTTAGCCTGGCCACGCGGAACCCCTATTTGT	1006
LacZ	TTACGGGGGTATGACGGCAAGTCGTTTTACAACGTCGT	GGCGGTCACGACTTTGCGAATTATTTTTGACACCAGACCAAC	3088
